# Comparison of a Scheimpflug Camera and Optical Coherence Tomography in Evaluating Keratoconic Eyes Post Keratoplasty

**DOI:** 10.3390/jcm14010238

**Published:** 2025-01-03

**Authors:** Anna Maria Gadamer, Piotr Miklaszewski, Dominika Janiszewska-Bil, Anita Lyssek-Boroń, Dariusz Dobrowolski, Edward Wylęgała, Beniamin Oskar Grabarek, Katarzyna Krysik

**Affiliations:** 1 Department of Ophthalmology, St. Barbara Hospital, Trauma Centre, 41-200 Sosnowiec, Poland; piotrmiklaszewski94@gmail.com (P.M.); dominika.bjaniszewska@gmail.com (D.J.-B.); anitaboron3@gmail.com (A.L.-B.); dardobmd@wp.pl (D.D.); kkrysik@gmail.com (K.K.); 2 Department of Ophthalmology, Faculty of Medicine, Academy of Silesia, 40-555 Katowice, Poland; 3 Collegium Medicum, WSB University, 41-300 Dabrowa Gornicza, Poland; bgrabarek7@gmail.com; 4 Department of Ophthalmology, District Railway Hospital, 40-760 Katowice, Poland; wylegala@gmail.com; 5 Department of Ophthalmology, Faculty of Medicine, Medical University of Silesia, 40-555 Katowice, Poland

**Keywords:** keratoconus, OCT, CCT, TCT

## Abstract

**Background/Objective:** The aim of this retrospective study was to compare corneal parameters and compliance using a Pentacam HR–Scheimpflug (Pentacam HR) and a swept-source OCT Casia (Casia) in keratoconus (KC) patients post penetrating keratoplasty (PKP) and KC patients without PKP, as well as a control group. Pachymetry measurements were also analyzed using a spectral domain OCT Solix (OCT Solix), Pentacam HR, and Casia. **Methods:** The study included 71 patients (136 keratoconic eyes; group A), 86 eyes with KC post-PKP (group B), 50 eyes with KC without PKP (group C), and 52 control participants (104 eyes). All participants were adults, Polish Caucasian, and met specific inclusion criteria. Patients with ophthalmological or systemic diseases, cognitive impairment, or pregnancy were excluded. Corneal parameters were measured using two devices (Casia and Pentacam HR), while pachymetry was assessed with three devices (Casia, Pentacam HR, and OCT Solix), with the inter-device agreement and group differences analyzed. **Results:** Significant differences (*p* < 0.05) were found across all groups. The post-PKP KC eyes showed significant differences in all front parameters and K2 and Astig. back, while the non-PKP KC eyes showed differences in the K1 back (*p* = 0.025). The controls displayed differences in all parameters except front astigmatism (*p* = 0.61). The Pentacam HR overestimated the thinnest corneal thickness (TCT) compared to the OCT Casia across groups. The inter-device agreement was excellent for the anterior parameters (ICC > 0.9) but good for the posterior parameters and TCT. **Conclusions:** This study highlights significant variability in corneal and pachymetry measurements across devices, with OCT Casia providing more consistent and clinically reliable results than Pentacam HR. Clinicians should exercise caution when using these devices interchangeably, particularly for posterior parameters and TCT.

## 1. Introduction

Keratoconus (KC) is, in most cases, degenerative corneal ectasia characterized by progressive corneal thinning and structural changes in the stroma, resulting in irregular astigmatism, myopia, and potential corneal scarring [1]. Although its etiology remains unclear, KC has been linked to environmental, genetic, and systemic factors, including eye rubbing, atopic diseases, and connective tissue disorders [2,3].

KC typically manifests during puberty, with progression until the third or fourth decade of life, affecting vision quality and necessitating timely diagnosis and management [4,5]. The management of KC spans from spectacles and contact lenses in the early stages to surgical interventions in advanced cases. Corneal transplantation, including penetrating keratoplasty (PKP) and deep anterior lamellar keratoplasty (DALK), remains the definitive treatment for severe KC, with approximately 10–15% of patients requiring surgical correction [6,7]. The accurate assessment of corneal parameters is critical for diagnosing early KC, monitoring disease progression, and evaluating post-transplant outcomes. Advances in imaging technology, such as Scheimpflug tomography (e.g., Pentacam HR) and swept-source optical coherence tomography (SS-OCT, e.g., Casia), have enhanced clinicians’ ability to evaluate corneal parameters with precision [8,9].

Despite widespread use, these imaging systems often yield varying results for corneal thickness, curvature, and astigmatism. The implications of these discrepancies are particularly significant in post-PKP eyes, where structural changes influence imaging accuracy [10]. While previous studies have evaluated imaging system performance in KC, a comprehensive analysis comparing pachymetric and keratometric parameters across multiple devices in post-PKP eyes remains lacking [9,10]. Understanding the reliability and limitations of these devices in this context is crucial for optimizing post-surgical evaluation and management.

This study addresses this gap by performing a comparative analysis of Pentacam HR and Casia in KC patients post-PKP, KC patients without PKP, and a control group. Additionally, it evaluates pachymetry using OCT Solix. By providing a detailed assessment of device agreement and measurement variability, this research aims to guide clinicians in selecting appropriate imaging modalities for post-PKP keratoconic eyes.

## 2. Materials and Methods

### 2.1. Ethics

This retrospective study was conducted with the approval of the Ethics Committee operating at the Academy of Silesia, Katowice, Poland, No. 24/KB/AŚ/04/2024, obtained on 3 April 2024, and in accordance with the principles of the Declaration of Helsinki. Written informed consent was obtained from all participants.

The study was performed at the Ophthalmology Department of Saint Barbara Hospital, Trauma Center, Sosnowiec, Poland.

### 2.2. Subjects

The study included 71 patients (136 keratoconic eyes; group A), 86 eyes with KC post-PKP (group B), 50 eyes with KC without PKP (group C), and 52 control participants (104 eyes) between January 2018 and December 2022. The operations were performed by two experienced surgeons in corneal transplantation (Katarzyna Krysik and Dariusz Dobrowolski). In total, 65 patients had diagnosed keratoconus in both eyes, with 6 people having it unilaterally.

#### 2.2.1. Characteristics of the KC Patients Without PKP

This group included 50 eyes, evenly distributed between the left (23) and right (27). Of these, 13 eyes (26%) belonged to female participants with a mean age of 37.69 ± 12.97 years, while 37 eyes (74%) were from male participants with a mean age of 34.59 ± 11.73 years. The inclusion criteria required participants to provide written informed consent, be over 18 years old, have a diagnosis of keratoconus (KC) without PKP, and be of Polish Caucasian descent. The exclusion criteria included the refusal to participate, being under 18 years of age, absence of a keratoconus diagnosis, cognitive impairment, pregnancy, other corneal pathologies besides KC, or ophthalmological conditions such as glaucoma, retinal, choroidal, or optic nerve diseases, as well as cardiovascular conditions.

#### 2.2.2. Characteristics of the KC Patients with PKP

This group comprised 86 eyes that underwent PKP, including 33 left eyes, 23 right eyes, and 30 eyes (both left and right) from the same participants. Of these, 25 eyes (29.07%) were from female participants with a mean age of 38.88 ± 11.44 years, and 61 eyes (70.93%) were from male participants with a mean age of 35.26 ± 10.54 years. The inclusion criteria required participants to provide informed consent, be over 18 years old, have a diagnosis of keratoconus with PKP, and be of Polish Caucasian descent. The exclusion criteria included the refusal to participate, being under 18 years of age, the absence of a keratoconus diagnosis, undergoing a different type of corneal transplantation, pregnancy, other corneal pathologies aside from KC, or ophthalmological conditions such as glaucoma, retinal, choroidal, or optic nerve diseases, as well as cardiovascular conditions.

#### 2.2.3. Characteristics of the Control Group

The control group consisted of healthy adults without any known ophthalmological or systemic conditions. The participants were exclusively Polish Caucasians to reduce potential confounding variables. This group included 52 individuals (104 eyes), with 16 females contributing 32 eyes (30.77%) and a mean age of 38.03 ± 8.84 years and 36 males contributing 72 eyes (69.23%) with a mean age of 35.88 ± 9.15 years.

### 2.3. Ophthalmic Examination and Comparative Imaging Analysis

All participants underwent a comprehensive ophthalmic examination, which included best-corrected distance visual acuity (BCVA) assessment using the Snellen chart, intraocular pressure measurement using the i-care tonometer, slit-lamp biomicroscopy, and fundus examination under pupil dilation with 1% Tropicamide.

The corneal parameters were measured using two devices (Casia and Pentacam HR), while pachymetry was assessed with three devices: Casia, Pentacam (Pentacam HR, Oculus, Wetzlar, Germany), and OCT Solix. These devices acquired images by rotating a 475 nm slit-light source along the optical axis and capturing 25 to 100 slit images, utilizing Swept-Source technology. The inter-device agreement and group differences were analyzed. Three consecutive series of measurements were taken for each participant. The “best result” was determined based on the quality and consistency of the measurements as indicated by device-generated quality indices, such as the signal-to-noise ratio or imaging clarity. The measurements with artifacts or poor reproducibility were excluded. This approach ensured the selection of reliable and accurate data for further comparative analysis. Postoperative measurements were taken one year after PKP.

### 2.4. Optical Coherence Tomography

Swept-Source Optical Coherence Tomography (Casia SS-1000, Tomey, Nagoya, Japan) uses swept-source technology based on 3D Fourier-domain OCT with a light source of 1310 nm wavelength. The system takes 30,000 A-scans per second. The lateral resolution is 30.0 mm, and the axial resolution is 18.0 mm. Tissue scans are 16.0 mm in diameter and 6.0 mm deep. The software automatically analyzes the recorded images and provides various corneal maps, as well as a quantitative and qualitative anterior segment evaluation [11,12,13].

The Pentacam HR–Scheimpflug imaging system (Pentacam HR, Oculus, Wetzlar, Germany) scans are obtained by rotating around the optical axis from zero to 180° along with a monochromatic 475 nm UV-free slit-light source. Depending on the setting, the camera captures from 25 to 100 slit images.

This device is now widely used in daily clinical practice in ophthalmology [11,14,15,16].

Spectral Domain Optical Coherence Tomography (SOLIX, Optovue, Fremont, CA, USA) operates at 120,000 A-scans per second with the split spectrum amplitude–decorrelation angiography algorithm. The system is intended for in vivo imaging, cross-sectional and the three-dimensional imaging, and the measurement of the anterior and posterior ocular structures, including the retina, retinal nerve fiber layer, ganglion cell complex, optic disk, cornea, corneal epithelium and stroma, pachymetry, and anterior chamber of the eye. SOLIX has an integrated reference database that enables comparison of the measurements to this database of known normal subjects [17,18,19].

### 2.5. Statistical Analysis

The data were analyzed using R statistical software (version 4.4.00). Continuous variables were presented as medians with interquartile ranges (Q1–Q3) and minimum–maximum values. The normality of data distribution was assessed using the Shapiro–Wilk test. Differences between devices were evaluated using the Wilcoxon signed-rank test for pairwise comparisons and the Friedman two-way analysis of variance with Bonferroni correction for comparisons among three devices.

The agreement between devices was assessed using the intraclass correlation coefficient (ICC), interpreted as follows: poor agreement (ICC < 0.5), moderate agreement (ICC 0.51–0.7), good agreement (ICC 0.71–0.9), and excellent agreement (ICC > 0.9). The results were visualized using scatter plots, which included regression lines, ICC values, and the line of perfect agreement (Y = X). Bland–Altman plots were also employed to illustrate the mean differences and limits of agreement (±2 standard deviations).

For categorical variables, group differences were analyzed using the Chi-square test, while continuous variable differences across groups were assessed using one-way ANOVA or equivalent nonparametric tests when the data violated the normality assumptions. A *p*-value < 0.05 was considered statistically significant, with statistical significance consistently evaluated at this threshold.

## 3. Results

### 3.1. Patient Characteristics

The demographic and clinical characteristics of the study participants are summarized in Table 1. The distribution of gender and age across the four groups (A, B, C, and control) was analyzed. Group A included all eyes with keratoconus, while group B focused on eyes with keratoconus post-PKP. Group C consisted of keratoconus cases, with the control group comprising healthy eyes. Statistical comparisons were performed using the Chi-square test for gender distribution and one-way ANOVA for age, with no significant differences observed among the groups (*p* > 0.05).

### 3.2. Analysis of the Corneal Parameters

The corneal parameters, including the anterior and posterior keratometry at steep and flat meridians, astigmatism, and average keratometry, were measured using Casia and Pentacam HR devices. These measurements were compared between the keratoconus groups and the control group. Table 2 presents the corresponding corneal parameters measured using the Pentacam HR and Casia. The results for the corneal parameters for Groups A and B are shown in Table 3 and Table 4.

The measurements for the corneal front and back parameters demonstrated variations across the groups.

For Group A, the differences in corneal front parameters, such as K1 and Km, were statistically significant (*p* < 0.05), and for the corneal back, the Km, K2, and astigmatism also showed statistical significance. The Casia device yielded higher values for the corneal parameters compared to the Pentacam HR, except for astigmatism where the Pentacam HR reported slightly greater values. In group B, all the corneal front parameters were statistically significant, with K2 approaching borderline significance (*p* = 0.049). For the corneal back, significant differences were found in the K2 and astigmatism. On the other hand, group C revealed no statistically significant differences except for the K1 cornea back (*p* = 0.025).

In the control group, all parameters except for the anterior astigmatism (*p* = 0.61) were statistically significant (*p* < 0.001). The measurements using the Pentacam HR were generally lower than the Casia for K1, K2, and Km, with the exception of the astigmatism for keratoconic eyes, where the Pentacam HR reported greater values. In keratoconic eyes after PKP, higher astigmatism was observed compared to keratoconic eyes without PKP. This could be attributed to the influence of the sutures used in the PKP procedures. The astigmatism was lowest in the control eyes, and the percentage difference between devices was highest for the astigmatism due to the narrow range of results. The intraclass correlation coefficient (ICC) for groups B and C exceeded 0.9, indicating excellent agreement for the K1, K2, and Km cornea front and the K1 and Km cornea back. For the K2 cornea back, the ICC was approximately 0.9 for group B and higher than 0.9 for group C. In the control eyes, the ICC values also exceeded 0.9 for the K1, K2, and Km cornea front, suggesting that the differences in measurements were primarily due to variations in the corneal parameters rather than discrepancies between devices.

**Figure 1:**
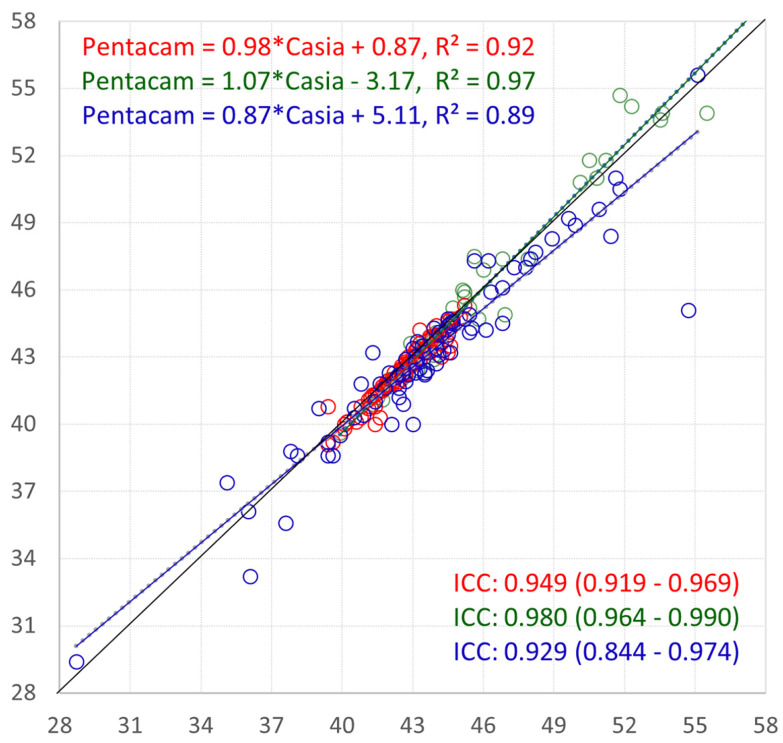
Linear regression with ICC values with 95% CI for K1/Kf cornea front. Keratoconic eyes without PKP: green; keratoconic eyes after PK: blue; control eyes: red.

**Figure 2:**
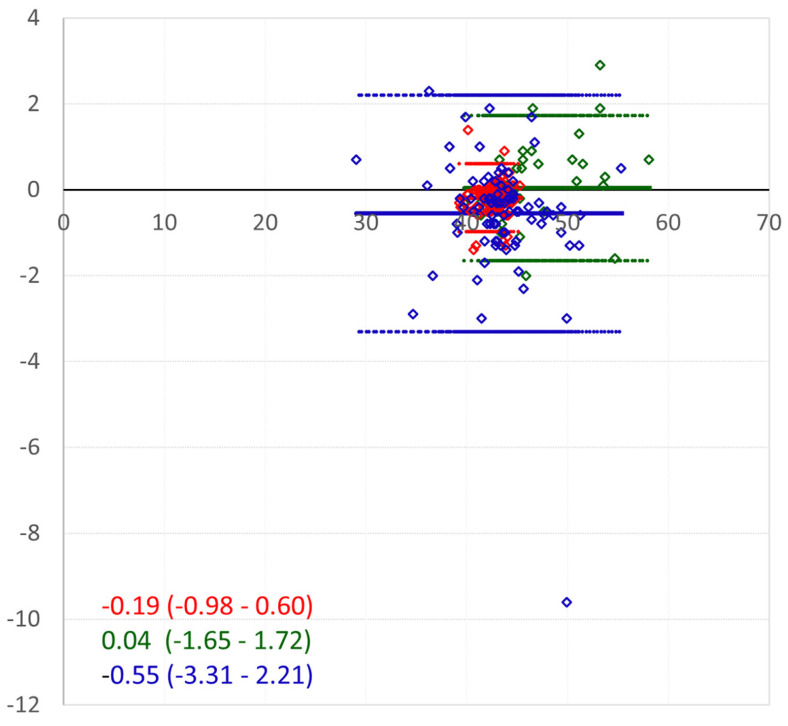
Bland–Altman plot for K1/Kf cornea front. Keratoconic eyes without PKP: green; keratoconic eyes after PKP: blue; control eyes: red. The *X*-axis represents the mean of the measurements, and the *Y*-axis represents their differences. The range of the mean difference is presented as ±2 standard deviations.

Figure 1 and Figure 2 illustrate the agreement between devices for the K1/Kf cornea front across the groups. Figure 1 presents the linear regression analysis with the ICC values and 95% confidence intervals, while Figure 2 displays the Bland–Altman plots showing the mean differences and ±2 standard deviations.

### 3.3. Analysis of CCT and TCT Parameters for Both Groups

The compliance for the central corneal thickness (CCT) and the thinnest corneal thickness (TCT) measurements using the three devices for Groups A and B is detailed in Table 5 and Table 6. The results obtained from the devices were mostly similar, although minor differences were observed across groups. For group A, the differences in the CCT measurements between the Pentacam HR and OCT Solix were statistically significant. For the TCT, significant differences were noted for each pair of devices. The Pentacam HR showed higher results for both the CCT and TCT compared to the Casia, while the OCT Solix reported the lowest measurements. For group B, statistically significant differences were observed in the CCT measurements between the Pentacam HR and OCT Solix and between the OCT Solix and Casia. For the TCT, significant differences were present for all device comparisons, with the Pentacam HR reporting the highest results and the OCT Solix the lowest. In group C, no significant differences were found for the CCT (*p* = 0.821), but statistically significant differences were observed for the TCT (*p* < 0.001). The OCT Solix yielded lower TCT results compared to the Pentacam HR and Casia, while it reported higher CCT results. The Pentacam HR consistently provided the highest TCT values.

**Figure 3:**
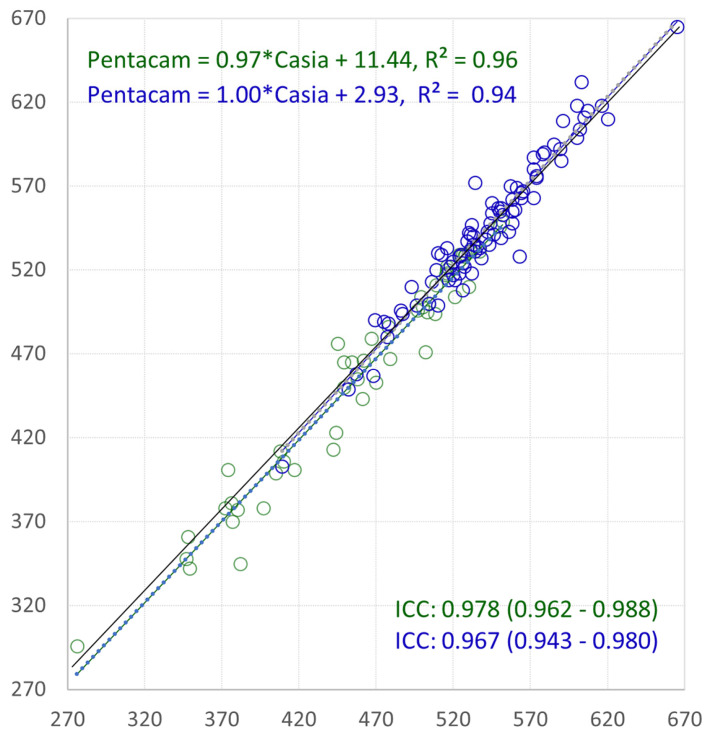
Linear regression with ICC values with 95% CI for the central corneal thickness (CCT) Pentacam HR versus Casia. Keratoconic eyes without PKP: green; keratoconic eyes after PKP: blue.

**Figure 4:**
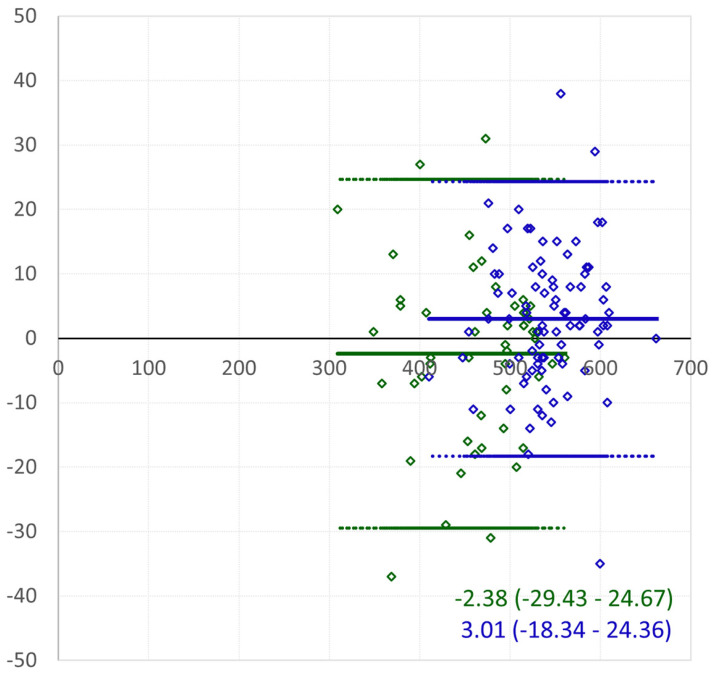
Bland–Altman plot for central corneal thickness (CCT) Pentacam HR versus Casia. Keratoconic eyes without PKP: green; keratoconic eyes after PKP: blue. The *X*-axis represents the mean of the measurements, and the *Y*-axis represents their differences. The range of the mean difference is presented as ±2 standard deviations.

Figure 3 and Figure 4 display the agreement in the CCT measurements between the Pentacam HR and Casia for keratoconic eyes with and without PKP, using linear regression and Bland–Altman analysis, respectively.

The ICC for the CCT was highest between the Pentacam HR and Casia, exceeding 0.9 and indicating excellent agreement. For keratoconic eyes without PKP, the ICC values for each device pair exceeded 0.9 for the CCT and 0.85 for the TCT. In keratoconic eyes after PKP, the ICC for the CCT was lowest (0.778) when comparing the Pentacam HR and OCT Solix, although it still indicated good agreement.

The ICC for the CCT was the highest between the Pentacam HR and Casia (>0.9 excellent agreement). For keratoconic eyes without penetrating keratoplasty, the ICC was >0.9 for each device, and for the TCT, it was >0.85. The ICC for the CCT for keratoconic eyes after PKP obtained the lowest results (0.778) when comparing the Pentacam HR to the OCT Solix, which was still good agreement.

## 4. Discussion

Exact and accurate corneal parameters are very important in clinical practice, especially in cornea diseases: firstly, to diagnose keratoconus, assess its progression, and manage it; secondly, to monitor the corneal parameters before and after transplantation. It is crucial to choose the appropriate device and analyze the parameters if we know that some devices may overestimate or underestimate the results. Often, corneal topographies cannot be used interchangeably, and different results could be obtained through using different devices. In our research, the results between different devices were mostly similar. We interpreted the device agreement in detail across different patient groups. To the best of our knowledge, no previous research has compared, in detail, the corneal parameters on such a large number of patients with keratoconus after PKP; in addition, no previous research has compared the CCT and TCT in keratoconic patients using these three devices. In our research, we determined ICC > 0.9 for the K1, K2, and Km cornea front for each group. These results were similar to Ghassemi et al. [20]. In the normal and keratoconus groups, there was perfect agreement between the Pentacam HR and CASIA2 for the anterior Ks and Kf.

Good agreement between two devices was presented by Ghoreishi et al. [21]. The authors found perfect agreement in normal corneas for all anterior cornea keratometry; the level of agreement in the KC cases ranged from moderate to strong. In addition, the posterior keratometry and corneal cylinder mostly indicated good agreement. There were statistically significant differences between two devices for the K1, K2, and Km anterior and posterior cornea for the control group, as well as in our research. The Pentacam HR obtained slightly lower corneal results, except for astigmatism for the control and KC groups, which was similar to our results. Lee et al. [22] investigated post-refractive surgery and normal eyes using dual rotating Scheimpflug–Placido, swept-source optical coherence tomography, and Placido–scanning-slit systems; they found that the agreement of the anterior keratometry and pachymetry between those three devices was high in both groups (ICC > 0.9). According to our study, the ICC between devices was better for the anterior parameters than the posterior measurements and astigmatism. The differences in measurements for each of our group were small, which indicates that both devices are quite consistent. However, for most of the parameters, the differences were statistically significant. The measurements obtained by the Casia and HR were mostly the same (*p* > 0.05) only for group C.

Szalai et al. [23] found significant differences in the anterior segment Fourier-domain AS-OCT and high-resolution Scheimpflug imaging measurements between a normal and keratoconus group. Bland–Altman plots showed good agreement between the measurements of the two devices. In their study, every measured parameter differed significantly between instruments, except the anterior astigmatism and posterior steep K results. In our research, the anterior astigmatism for group A and the control eyes and the anterior and posterior astigmatism for group C were not different. Viswanathan et al. [24] reported that the Pentacam HR measured significantly greater keratometry readings in the flattest and steepest meridians in normal and keratoconic eyes. In this study, they compared the Pentacam HR and a combined Placido–optical coherence tomography device (Visante OMNI). Both devices demonstrated excellent repeatability and reproducibility. Contrary to this, in our study, we found that the Pentacam HR showed lower results for the corneal parameters than the Casia. The Pentacam HR gave higher results for astigmatism for keratoconic eyes, and the differences were statistically significant. The different results may be due to the fact that, in our study, we included patients with keratoconus after penetrating keratoplasty, and we used other devices. In our research, the CCT in each group was similar, using Casia and Pentacam HR. There were no statistically significant differences. In contrast, the TCT differences were statistically significant for each group. Regarding this parameter, the Pentacam HR overestimated the results compared with other devices.

In the literature, there are conflicting results as to which devices underestimate and which may overestimate the CCT measurements. Kumar et al. [25] found that the CCT measured via the SS-OCT was overestimated (about 7 μm) compared to the Pentacam HR. Grewal et al. [26] compared the CCT using the Pentacam HR and AS-OCT in normal and in keratoconic eyes. They found that the AS-OCT measured significantly higher readings (mean difference about 2 μm) than the Pentacam HR in keratoconic eyes. The Pentacam HR’s tendency to underestimate the results was also mentioned in other articles [27]. On the other hand, some studies have shown lower CCT measurements with the AS-OCT when compared to ultrasound pachymetry or Orbscan [28]. The results for normal subjects were also different: in some articles, the mean CCT using the Pentacam HR and Casia were similar [29]; in others, these devices were not interchangeable [11,26]. In our study, there were no statistically significance differences for the CCT between the Pentacam HR and Casia in groups A, B, and C.

Interestingly, in our study, in every group, the Pentacam HR showed the highest results for the thinnest point. The Pentacam HR also overestimated the TCT in other studies [24,30]. Ghassemi et al. [20] showed that the TCT measurements with the Pentacam HR were thicker than those analyzed with the CASIA2. Szalai et al. [23] demonstrated that the thinnest point of the cornea was overestimated using a Pentacam HR in the normal and keratoconus groups, which corresponded to our results. On the other hand, Seiler et al. [31] showed that the thinnest pachymetry was approximately 5 µm thinner when measured using the Pentacam HR than the OCT MS-39. There were statistically significant differences in the TCT measurements between the Pentacam HR and OCT Solix in each group (*p* < 0.001). The SD-OCT Solix obtained higher results for the CCT compared to the Casia and Pentacam HR only in group C, but those results were not statistically significant. In groups A and B, the CCT showed the lowest results. In contrast to our study, Kumar et al. [12] compared the CCT measurements in keratoconic eyes using four devices: HHSD-OCT, Pentacam, Orbscan IIz, and SS-OCT. This research showed that the HHSD-OCT overestimated the CCT, and there was good correlation between the measurements obtained from the other three devices. Li et al. [32] compared the SD AS-OCT (REVO-NX) and Pentacam-AXL; they found that both devices demonstrated good repeatability but poor agreement across the AL, ACD, CCT, and TCT measurements. The results of this study indicate a better repeatability using Scheimpflug imaging compared to AS-OCT. We also recommend not to use the Pentacam HR and OCT Solix interchangeably. In our study, the ICC for the CCT and TCT was mostly the lowest when comparing the OCT Solix to the Pentacam HR. The results obtained by the different devices in our research were similar. We demonstrated that, in divided groups, the differences were low but sometimes statistically significant. The devices are not interchangeable. In our study, the results for the corneal parameters obtained using the Pentacam HR were mostly lower than using the Casia.

The measurements using the Scheimpflug camera can be different because this device measures the corneal thickness between the air–tear film interface and the posterior corneal surface, and the results are affected by the tear film quality [33]. We compared the central corneal thickness measurements by rotating the Scheimpflug camera ultrasonically. We also noticed that the tests performed using the OCT Casia took less time and were easier for our patients. In the available literature, many authors suggest choosing the Casia over the Pentacam HR. Szalai et al. [34] also suggested choosing SS-OCT in patients who had undergone corneal transplantation. Chan et al. [35] concluded that Casia might be preferred because of the better repeatability of measurements. In addition, Feldman et al. [30] found that the Casia had better repeatability and reproducibility compared to the Pentacam HR for both the corneal curvature and pachymetry maps. Ortiz-Toquero et al. [36] found the accuracy of the Pentacam HR and Casia SS-1000 to be good in healthy subjects and patients with keratoconus, but the Casia presented better repeatability and reproducibility in all parameters in all groups compared to the Pentacam HR. Moreover, they found that the reliability of the intrasession repeatability and the intersession reproducibility worsened with increasing keratoconus severity. Seiler et al. [31] demonstrated that a further progressed keratoconus stage was significantly associated with increased measurement errors and resulted in worse repeatability.

## 5. Conclusions

In conclusion, the results of our study show that the results obtained using different devices were mostly similar; however, in some parameters, the differences were statistically significant. Sometimes small differences may be clinically significant and should be taken into account in clinical practice. Clinicians should be aware of device limitations.

The limitation in our research is that there was no division of keratoconic eyes according to the severity of the disease; on the other hand, this may have resulted in an insufficient number of subjects for statistical analysis.

## Figures and Tables

**Table 1:** Demographic and clinical characteristics of the patients across the study groups.

Group	A	B	C	Control	*p*-Value
n (eyes)	136	86	50	104	
gender females	38 eyes (28.17%)	25 eyes (29.07%)	13 eyes (26%)	32 eyes (30.77%)	0.439 ^1^
gender males	98 eyes (71.83%)	61 eyes (70.93%)	37 eyes (74%)	72 eyes (69.23%)	
age females mean ± SD	38.11 ±11.16	38.88 ± 11.44	37.69 ± 12.97	38.03 ± 8.84	0.718 ^2^
age males mean ± SD	34.93 ± 9.25	35.26 ± 10.54	34.59 ± 11.73	35.88 ±9.15	0.751 ^2^

A, all eyes with keratoconus (136 eyes); B, eyes with keratoconus after PKP (86 eyes); C, eyes with keratoconus; n, number of cases. Data are presented as the number of cases (percentage) or the mean ± standard deviation; ^1^, *p*-value of the Chi-square test; ^2^, *p*-value of the one-way ANOVA variance analysis.

**Table 2:** Corresponding corneal parameters for Pentacam HR versus Casia.

PENTACAM HR	CASIA
K1 Cornea Front	Kf Anterior
K2 Cornea Front	Ks Anterior
Km Cornea Front	AvgK Anterior (Average K)
Astig. Cornea Front (Astigmatism)	Cyl Anterior (Cylinder)
K1 Cornea Back	Kf Posterior
K2 Cornea Back	Ks Posterior
Km Cornea Back	AvgK Posterior (Average K)
Astig. Cornea Back (Astigmatism)	Cyl Posterior (Cylinder)

**Table 3:** Results for corneal parameters (cornea front and back) for group A (all keratoconic eyes—136 eyes) for the Pentacam HR versus Casia.

Cornea Front				
	K1 Cornea	K2 Cornea	Km Cornea	Astig. Cornea
Casia	43.9 [42.58; 45.6]	47.65 [45.28; 50.13]	45.83 [43.99; 47.78]	3.3 [1.9; 5.08]
	28.7; 57.7	34.8; 60.9	31.75; 59.3	0.1; 9.3
Pentacam HR	43.5 [42.2; 45.2]	47.15 [44.98; 49.95]	45.25 [43.65; 47.88]	3.2 [1.8; 5.43]
	29.4; 58.4	35.4; 63.1	32.4; 60.35	0.3; 10.4
Dif	−0.3 [−0.8; 0.2]	−0.1 [−0.6; 0.33]	−0.2 [−0.6; 0.11]	0.1 [−0.3; 0.63]
	−9.6; 2.9	−6.4; 5.1	−5.75; 2.7	−7.1; 7.7
%DifPC	1.15 [0.49; 2.34]	1.04 [0.49; 1.82]	0.92 [0.44; 1.82]	14.64 [8.12; 28.24]
	0; 17.55	0; 12.26	0; 10.26	0; 80
*p* *	<0.001	0.063	<0.001	0.094
Cornea back				
	K1 Cornea	K2 Cornea	KM Cornea	Astig. Cornea
Casia	−6.4 [−6.7; −6]	−6.9 [−7.5; −6.6]	−6.65 [−7.15; −6.35]	0.5 [0.3; 0.8]
	−10; −5.5	−10.4; −5.7	−10.2; −5.6	0.1; 1.7
Pentacam HR	−6.3 [−6.7; −6]	−7.05 [−7.6; −6.6]	−6.65 [−7.16; −6.4]	0.6 [0.4; 1.1]
	−9.7; −5.2	−10.5; −5.7	−10.1; −5.65	0; 2.3
Dif	0 [−0.1; 0.2]	−0.1 [−0.23; 0]	−0.05 [−0.15; 0.05]	0.1 [0; 0.3]
	−0.7; 1	−0.8; 1.4	−0.55; 1.2	−1.4; 1.4
%DifPC	1.71 [0; 3.64]	2.67 [1.4; 4.62]	1.65 [0.77; 2.97]	25 [12.5; 50]
	0; 12.35	0; 16.67	0; 14.55	0; 100
*p* *	0.115	<0.001	0.011	<0.001

Median [Q1; Q3], min–max. %Dif PC = absolute percentage difference = 100% × |Pentacam; Casia|/|Max(Pentacam; Casia)|. * *p* for the Wilcoxon signed-rank test.

**Table 4:** Results for corneal parameters (cornea front and back) for group B (keratoconic eyes after PKP—86 eyes) for the Pentacam HR versus Casia.

**Cornea Front**				
	K1 Cornea	K2 Cornea	Km Cornea	Astig. Cornea
Casia	43.5 [42.15; 45.35]	47.85 [45.43; 49.7]	45.98 [44; 47.31]	3.7 [2.6; 5.4]
	28.7; 55.1	34.8; 57.5	31.75; 56.15	0.5; 9
Pentacam HR	43.2 [41.65; 44.58]	47.6 [45.23; 49.48]	45.25 [43.71; 47.06]	4.05 [2.43; 6]
	29.4; 55.6	35.4; 56.1	32.4; 55.85	0.4; 10.4
Dif	−0.4 [−1; −0.03]	−0.1 [−0.6; 0.3]	−0.23 [−0.75; 0.05]	0.2 [−0.3; 0.8]
	−9.6; 2.3	−6.4; 1.9	−5.75; 1.6	−3.8; 7.7
%DifPC	1.24 [0.68; 2.57]	1.06 [0.57; 1.79]	0.9 [0.36; 1.85]	13.71 [7.02; 28.04]
	0; 17.55	0; 11.27	0; 10.26	0; 80
*p* *	<0.001	0.049	<0.001	0.025
**Cornea back**				
	K1 Cornea	K2 Cornea	Km Cornea	Astig. Cornea
Casia	−6.35 [−6.58; −6.03]	−6.9 [−7.2; −6.6]	−6.6 [−6.84; −6.4]	0.5 [0.3; 0.9]
	−8.4; −5.5	−10.1; −6	−9.25; −5.75	0.1; 1.7
Pentacam HR	−6.3 [−6.6; −6]	−7.05 [−7.4; −6.7]	−6.65 [−6.9; −6.46]	0.7 [0.4; 1.18]
	−9; −5.2	−9.2; −6.2	−9.1; −5.8	0; 2.3
Dif	0 [−0.1; 0.1]	−0.15 [−0.3; 0]	−0.05 [−0.15; 0.05]	0.1 [0; 0.3]
	−0.7; 0.6	−0.7; 0.9	−0.55; 0.35	−1.4; 0.9
%DifPC	1.61 [0; 3.27]	2.76 [1.39; 4.48]	1.56 [0.76; 2.61]	25.83 [12.5; 50]
	0; 10.34	0; 12.9	0; 6.77	0; 100
*p* *	0.994	<0.001	0.114	<0.001

Median [Q1; Q3], min–max. %Dif PC = absolute percentage difference = 100% × |Pentacam; Casia|/|Max(Pentacam; Casia)|. * *p* for the Wilcoxon signed-rank test.

**Table 5:** Compliance for CCT and TCT measurements for group A (all keratoconic eyes—136 eyes) using three devices.

	CCT	TCT
Casia	524.5 [478; 551]	502 [454.75; 528]
	276; 665	241; 651
Pentacam HR	526 [487.5; 553.25]	507.5 [472; 537.25]
	296; 665	274; 629
OCT Solix	523 [481.5; 550.25]	497.5 [455; 534.25]
	323; 691	237; 641
DifPC	2 [−5; 8]	8 [1.75; 17]
	−37; 38	−150; 164
DifSC	−4 [−8; 4]	−5 [−8.25; −1]
	−47; 167	−107; 208
DifSP	−3 [−12.25; 5]	−13 [−22; −3.75]
	−76; 163	−87; 224
%DifPC	1.33 [0.6; 2.4]	2.16 [0.9; 3.88]
	0; 9.69	0; 35.12
%DifSC	1.32 [0.75; 2.77]	1.52 [0.88; 2.33]
	0; 24.17	0; 38.45
%DifSP	2.07 [0.67; 4.01]	2.86 [1.18; 4.55]
	0; 23.59	0; 41.4
*p* *	0.002	<0.001
*p* # for DifPC	0.642	<0.001
*p* # for DifSC	0.094	<0.001
*p* # for DifSP	0.002	<0.001

Median [Q1; Q3], min–max. * *p* for Friedman test. # adjusted (Bonferroni method) *p* for nonparametric post hoc tests. %Dif_PC_ = 100% × |Pentacam HR; Casia|/|Max(Pentacam HR; Casia)|. %Dif_SC_ = 100% × |OCT Solix; Casia|/|Max(OCT Solix; Casia)|. %Dif_SP_ = 100% × |OCT Solix; Pentacam HR|/|Max(OCT Solix; Pentacam HR)|. | |—the absolute value.

**Table 6:** Compliance for CCT and TCT measurements for group B (keratoconic eyes after PKP—86 eyes) using three devices.

	CCT	TCT
Casia	539.5 [520; 564]	518 [488.5; 543.75]
	409; 665	345; 651
Pentacam HR	540.5 [520.5; 568.5]	526 [499.25; 553.75]
	403; 665	323; 629
OCT Solix	538 [516.75; 570]	513.5 [492.25; 543.5]
	418; 691	345; 641
DifPC	2.5 [−3; 9.75]	6.5 [−0.5; 17]
	−35; 38	−150; 116
DifSC	−5 [−9; 1.75]	−6 [−9.75; 0]
	−47; 167	−107; 118
DifSP	−5 [−16; 1.75]	−10 [−20.75; −2]
	−76; 163	−87; 212
%DifPC	1.32 [0.59; 2.15]	2.11 [0.77; 3.72]
	0; 6.64	0.18; 31.71
%DifSC	1.28 [0.7; 2.27]	1.54 [0.95; 2.74]
	0; 24.17	0; 21.77
%DifSP	2.03 [0.67; 3.71]	2.46 [0.82; 4.16]
	0; 23.59	0; 39.63
*p* *	<0.001	<0.001
*p* # for DifPC	0.280	0.001
*p* # for DifSC	0.007	0.002
*p* # for DifSP	<0.001	<0.001

Median [Q1; Q3], min–max. * *p* for Friedman test. # adjusted (Bonferroni method) *p* for nonparametric post hoc tests. %Dif_PC_ = 100% × |Pentacam HR; Casia|/|Max(Pentacam HR; Casia)|. %Dif_SC_ = 100% × |OCT Solix; Casia|/|Max(OCT Solix; Casia)|. %Dif_SP_ = 100% × |OCT Solix; Pentacam HR|/|Max(OCT Solix; Pentacam HR)|. | |—the absolute value.

## Data Availability

The original contributions presented in this study are included in the article; further inquiries can be directed to the corresponding author.
